# Quadricuspid Aortic Valve: Report of Two Cases and Brief Review

**DOI:** 10.1155/2019/7835287

**Published:** 2019-04-09

**Authors:** Oreoluwa Oladiran, Ifeanyi Nwosu, Rashmi Dhital, Gbujie Ezioma

**Affiliations:** ^1^Reading Hospital, Tower Health System, West Reading, Pennsylvania, USA; ^2^Leighton Hospital NHS Trust, Crewe, Cheshire, UK; ^3^Saint Peter's University, New Brunswick, New Jersey, USA

## Abstract

Quadricuspid aortic valve (QAV) is a rare congenital cardiac defect characterized by the presence of four aortic valve leaflets of equal or varying sizes. Even rarer is its clinical presentation with aortic stenosis. Diagnosis of QAV could be challenging but is of great importance as patients often present with progressive aortic regurgitation. We present 2 cases of QAV presenting differently: one with aortic stenosis requiring valve replacement and the other with aortic regurgitation requiring close monitoring.

## 1. Case 1

62-year-old woman with medical history significant for aortic stenosis and chronic atrial fibrillation presented to the emergency department with fatigue and progressively worsening shortness of breath with minimal exertion. She was asymptomatic at rest and denied chest pain, orthopnea, paroxysmal nocturnal dyspnea, leg swelling, presyncope, or syncope. She was incidentally found to have a systolic murmur during her pregnancy 20 years prior to this presentation. Notably, she had been offered aortic valve replacement in the past but declined.

Vital signs were normal with blood pressure of 110/60 mmHg, pulse rate of 79/min, temperature of 97.2°F, and respiratory rate of 16 breaths/min with normal oxygen saturation of 100% on ambient air. Physical examination was significant for irregularly irregular heart rhythm, and ejection systolic murmur was loudest in the aortic area with radiation to the carotids. Her lungs were clear to auscultation, and no pedal edema was noted. Electrocardiogram revealed atrial fibrillation with voltage criteria for left ventricular hypertrophy. Her most recent transthoracic echocardiogram revealed a thickened calcified aortic valve with decreased excursion with 4.6 m/s velocity suggesting a peak of 86 mmHg and mean of 36 mmHg suggestive of severe aortic stenosis. No other significant valvular abnormalities noted. Cardiac catheterization revealed widely patent coronary arteries.

**Figure 1:**
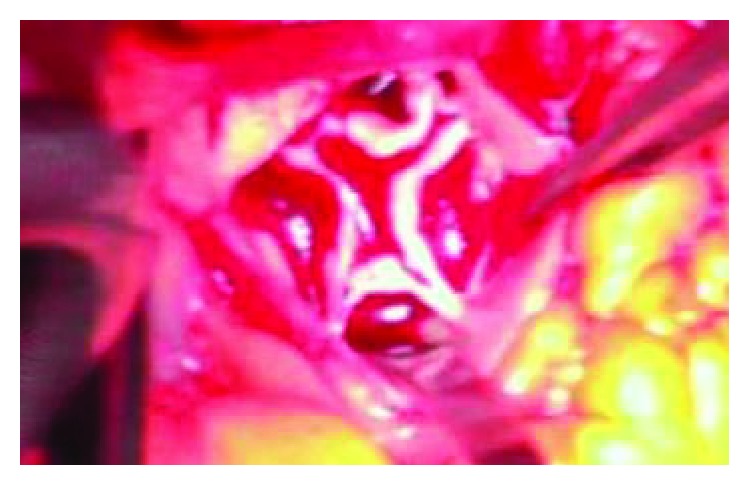
Intraoperative image showing quadricuspid aortic valve: Hurwitz type C/Nakamura type IV.

Based on the presence of worsening symptoms and the risk of sudden cardiac death, the decision was made to proceed with surgical aortic valve replacement. Given her history of chronic atrial fibrillation, she was also planned for left atrial appendage exclusion and Cox Maze IV procedure simultaneously with the aortic valve replacement. Intraoperative transesophageal echocardiogram revealed quadricuspid aortic valves confirmed during surgical exploration (Figure 1). The native stenotic quadricuspid aortic valve leaflets were excised, and a 21 mm Saint Jude Medical Trifecta valve was implanted. The left atrial appendage was excised, and Cox Maze IV procedure was performed. Postoperatively, she remained in junctional rhythm and underwent uneventful placement of the dual chamber pacemaker on postoperative day 3. She recovered without further complications and was discharged on the eight postoperative day.

## 2. Case 2

53-year-old female with past medical history of aortic regurgitation and hypertension presented to the cardiology office for routine follow-up. She denied chest pain, shortness of breath, orthopnea, dyspnea, or leg swelling. Physical examination revealed normal vital signs. Cardiac auscultation revealed diastolic murmur loudest at the 3rd left intercostal space. The remainder of physical examination was unremarkable. Transthoracic echocardiogram (TTE) 3 months earlier revealed a trileaflet aortic valve with moderate aortic insufficiency. TTE also noted a poorly defined subaortic membrane which prompted further assessment of valve anatomy by transesophageal echocardiogram (TEE) which confirmed the presence of quadricuspid aortic valve with severe aortic regurgitation from incomplete coaptation of the valve leaflets. Given that she was asymptomatic, we planned to continue surveillance by clinical and echocardiographic monitoring.

## 3. Discussion

The normal aortic valve is trileaflet and with the pulmonary valve represents the two semilunar heart valves. The leaflets, namely, right coronary, left coronary, and noncoronary cusps, are named based on their relationship to the coronary arteries [1]. Anatomical variations of the aortic valve have been documented notably, the unicuspid, bicuspid, and quadricuspid valves. Of these variants, the bicuspid valve is the commonest followed by unicuspid valve [2]. Quadricuspid aortic valve (QAV) is a rare congenital anomaly with documented incidence of 0.008% by autopsy, 0.043% by transthoracic echocardiogram, and 1% intraoperatively during aortic valve surgery [3, 4]. While both of our patients were female, it has been shown to have a slightly higher male preponderance [5] with mean age of presentation between 46 and 50 years. The embryology of QAV remains poorly understood, but it is believed to result from aberrant division of one of the three mesenchymal ridges that normally gives rise to three aortic valve cushions [6, 7]. Acquired heart diseases such as infective endocarditis and rheumatic fever may contribute to the development of a false QAV, but the absence of corpus arantii (nodules of semilunar cusps) helps to distinguish true congenital from an acquired QAV [8].

**Figure 2:**
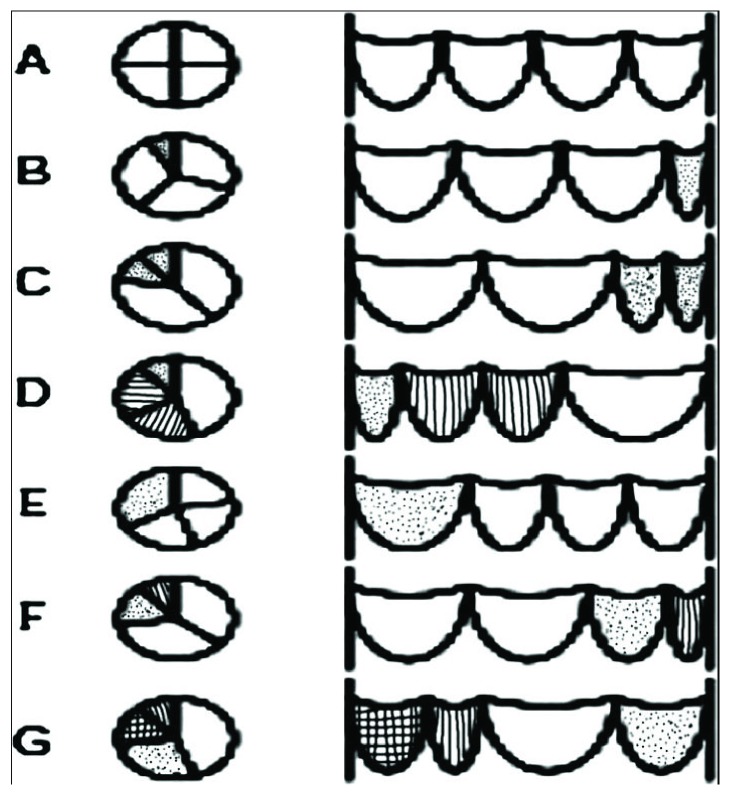
Hurwitz and Roberts classification of quadricuspid aortic valve.

**Figure 3:**
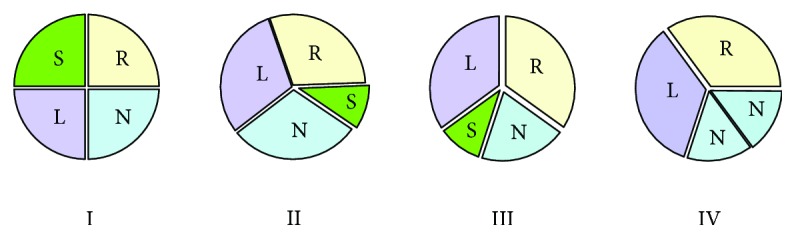
Nakamura et al. classification of quadricuspid aortic valve. L = left coronary cusp; N = noncoronary cusp; R = right coronary cusp; S = supernumerary cusp.

Hurwitz and Roberts described the seven (A–G; Figure 2) most common anatomic variants of QAV based on the size of the valve leaflets [9]. Type A has 4 equal cusps, type B has 3 normal cusp and a small accessory cusp, and type C has 2 large cusps and 2 small cusps. Types A and B represent about 73% of all the cases of QAV [10]. Other classification systems exist based on the position of the accessory cusps as described by Nakamura et al. [11] (Figure 3). In type I, the supernumerary cusp lies between the left and right coronary cusps. In type II, it lies between the right and noncoronary cusps. In type III, it lies between the left and noncoronary cusps, and in type IV, the supernumerary cusp cannot be identified as there are 2 equal sized smaller cusps. It is important to note that the position of the accessory cusp has no effect on the severity of the valvular insufficiency [4, 12]. Based on these classifications, our first patient had Hurwitz type C and Nakamura type IV while the second patient likely has Hurwitz type A and Nakamura type I quadricuspid aortic valve.

Quadricuspid aortic valve (QAV) usually presents as an isolated congenital anomaly, but other associated cardiac anomalies have been reported in about 18-32% of cases including aortic root dilatation [13], tetralogy of Fallot [14], patent ductus arteriosus [15], atrial and ventricular septal defects [16, 17], and anomalous origin of the coronary arteries [12].

Clinical presentation varies being mostly asymptomatic in the young, recall that both patients were asymptomatic until adulthood. The symptomatic patients will present with palpitations, dyspnea, fatigue, chest pain, and feature of congestive heart failure. Cases of ischemic stroke as an initial presentation has been reported [18]. Patients with QAV mostly present with aortic insufficiency and less commonly aortic stenosis [5]. More than half will present in adulthood with features of aortic insufficiency and more than 50% will require aortic valvular replacement in the sixth and seventh decade of life due to worsening aortic regurgitation [2, 19]. Infective endocarditis occurs in 1.4% of these patients [8] depending on the Hurwitz classification and its effect on dynamics of blood flow across the valve as mechanical stress on the abnormal valves [20] is thought to play a key role.

The routine use of echocardiography in suspected cases of valvular heart disease has allowed for early and accurate diagnosis of QAV. Although transthoracic echocardiography is often used in clinical settings, transesophageal echocardiography provides a more accurate assessment of valvular anatomy [8]. Magnetic resonance imaging and computerized tomography can also aid diagnosis, although not generally used solely for this purpose [18, 21]. In the absence of symptoms as described above, patients with QAV should be followed up closely. For symptomatic patients, treatment options range from valve repair to valve replacement. Repair may be feasible in some patients, but most will require valve replacement. Valve replacement, which could be surgical as in the first case described or transcatheter, is preferred in patients with severe aortic valvular dysfunction. Tricuspidalization is a preferred repair option in patients with Hurwitz types A, B, and C QAV [4].

## 4. Conclusion

Quadricuspid aortic valve is a rare congenital cardiac defect usually diagnosed incidentally. While most patients develop aortic regurgitation later in life, some as reported in the first case develop aortic stenosis while others remain asymptomatic. Although there are no guideline-based approach to management, it is prudent to regularly follow up these patients for early signs of aortic valve dysfunction.

## Supplementary Materials

Supplementary Materials Transthoracic echocardiogram (parasternal short axis view) clearly showing quadricuspid aortic valve.
